# Rapid Artificial Infestation Method for Assessing Fall Armyworm (*Spodoptera frugiperda*) Damage on Maize

**DOI:** 10.3390/insects17020136

**Published:** 2026-01-24

**Authors:** Caiyao Wu, Weiting Chen, Xinyu Guo, Gongwen He, Guiqin Yang, Lili Zhu, Juan Yao, Dagang Jiang

**Affiliations:** Guangdong Provincial Key Laboratory for the Development Biology and Environmental Adaptation of Agricultural Organisms, College of Life Sciences, South China Agricultural University, Guangzhou 510642, China; wucaiyao@stu.scau.edu.cn (C.W.); chenweit@126.com (W.C.); xinyuguo@stu.scau.edu.cn (X.G.); gongwenhe@stu.scau.edu.cn (G.H.); yangguiqin1120@stu.scau.edu.cn (G.Y.); lilizhu@scau.edu.cn (L.Z.); yaojuan@scau.edu.cn (J.Y.)

**Keywords:** *Spodoptera frugiperda*, transgenic maize, resistance evaluation, infestation protocols

## Abstract

The fall armyworm (*Spodoptera frugiperda*) is a globally invasive pest that threatens the yield of maize and other grain crops. Transgenic insect-resistant maize offers an effective management strategy; however, rigorous evaluation of resistance to it depends on rapid and standardized infestation protocols. We developed and benchmarked laboratory, screenhouse, and field methods for rapid resistance assessment using 1–4-day-old larvae (L1–L4) and maize whorl leaves, silks, and kernels as feeding substrates. In laboratory bioassays, five L2 on each leaf or silk treatment enabled resistance assessment on day 2 post-infestation, whereas two L1 per treatment on kernels supported evaluation on day 3. In screenhouse trials, infesting each plant with twenty L2 allowed reliable leaf-injury ratings on day 10. In field trials, thirty L3 per plant with assessment on day 12 produced better outcomes. Together, these protocols provide a detailed and adaptable framework that reduces costs, shortens evaluation timelines, and offers practical guidance for resistance assessment of transgenic maize across controlled and open environments.

## Simple Summary

The fall armyworm is a destructive pest that has spread globally, causing severe damage to crops such as maize. Scientists have developed genetically modified maize to resist this pest; however, assessing its effectiveness requires efficient and reliable evaluation methods. This study aimed to establish a simple and rapid assessment protocol suitable for laboratory, screenhouse, and field environments. We found that infestation of maize plants with a defined number of early-instar larvae allows accurate assessment of plant resistance within approximately 10–12 days. This method reduces costs and shortens the evaluation timeline, while providing practical and standardized operational guidance for evaluating insect-resistant transgenic maize.

## 1. Introduction

The fall armyworm (*Spodoptera frugiperda* [J. E. Smith]) is a highly destructive, polyphagous lepidopteran native to the tropical and subtropical Americas. It feeds on more than 350 plant species, including maize, rice, wheat, sorghum, soybean, and cotton [1,2]. In December 2018, *S. frugiperda* entered Yunnan Province from Myanmar and, owing to its strong migratory capacity and ecological plasticity, rapidly spread to 27 provinces, causing substantial maize yield losses in Southwest and South China. It has since become a major migratory pest that threatens China’s maize production and national food security [3,4].

Beyond chemical control, transgenic insect-resistant maize has become a key tactic against *S. frugiperda*. Since the first commercial approval of the Bt maize event Bt176 in the United States in 1996, hybrids expressing distinct insecticidal proteins (e.g., Cry1Ab, Cry1F) have been widely adopted to target lepidopteran pests such as the Asian corn borer (*Ostrinia furnacalis*) and the corn earworm (*Helicoverpa zea* Boddie) [5,6]. Among these, many Bt maize hybrids (e.g., those expressing Cry1F or the pyramided proteins Cry1A.105 and Cry2Ab2) have also demonstrated high efficacy against the subsequently invasive fall armyworm and, as a result, have become one of the primary control measures in countries such as the United States, Canada, and South America [7,8,9]. In China, insect-resistant maize events such as DBN9936 (*cry1Ab* + *epsps*) and Ruifeng 125 (*cry1Ab* + *cry2Aj* + *epsps*) have received production-use safety certificates and contribute to the management of this pest [10].

Robust resistance evaluation is indispensable during the research, deregulation, and stewardship phases of transgenic maize to identify transformation events suitable for commercialization. Laboratory bioassays of Rodriguez-Chalarca and colleagues [11] reported >90% mortality of *S. frugiperda* larvae on VT2P maize (expressing Cry1A.105 and Cry2Ab2) and VT3P maize (expressing Cry1A.105, Cry2Ab2, and Cry3Bb1). Zhao and colleagues [12] further showed that Bt maize DBN3601T carrying the *cry1Ab* and *vip3Aa19* transgenes caused 100% mortality of neonates by day 3 when they fed on leaves collected at different plant growth stages. Nevertheless, toxicity differs among Bt proteins (e.g., Cry1Ab, Cry1F, Vip3A), and expression levels and efficacy can vary across transformation events and genetic backgrounds [13,14]. These realities underscore the need for standardized, operational procedures for resistance evaluation in maize. However, systematic, detailed protocols for infestation and rating against *S. frugiperda* remain limited. For instance, the leaf damage rating methods proposed by Williams et al. [15] and by Toepfer et al. [16], respectively, lack standardized specifications for larval age and infestation density, which likely compromises the comparability of experimental results. Therefore, this study aims to establish rapid, accurate, and detailed laboratory and field infestation methods by analyzing the effects of infestation timing, larval density, and larval age on the degree of maize damage. Our goal is to deliver efficient, reliable protocols for resistance assessment that improve evaluation throughput while reducing research and development costs.

## 2. Materials and Methods

### 2.1. Maize Materials and S. frugiperda Colony

The transgenic maize hybrid Xianda 901ZL, developed by China National Seed Group Co., Ltd., Beijing, China, carries the transgenes *vip3Aa20*, *cry1Ab*, *epsps*, and *pat*, conferring insect resistance and herbicide tolerance; this hybrid has obtained biosafety certification in China. The non-transgenic control was the commercial hybrid Guidan 162, which does not contain insect-resistance traits and was purchased from the market.

*S. frugiperda* colony used in this study was obtained from Baiyun Industrial Co., Ltd. (Jiyuan, Henan, China). After three generations of laboratory rearing, larvae from the third generation were used for subsequent experiments. All rearing was conducted in programmable climate chambers at 26 ± 1 °C, a 16:8 h light–dark photoperiod, and 70–80% relative humidity. Eggs were incubated until hatching; neonates were reared in plastic boxes (29.3 × 19.3 × 9.6 cm) on an artificial diet. After reaching the third instar, larvae were transferred to 24-well plastic rearing plates (13 × 8.5 × 2.8 cm; one larva per well) and maintained individually until pupation. Newly emerged adults were held in rearing cages (40 × 40 × 40 cm) and supplied with 10% (*v*/*v*) honey solution for water and nutrients; egg masses were collected for subsequent trials.

The artificial diet followed Zhao et al. [12] with minor modifications: soy flour, wheat bran, brewer’s yeast, sorbic acid, and casein were weighed and mixed thoroughly. Separately, 20 g agar was dissolved by boiling in 1300 mL distilled water and the dry mixture was incorporated with stirring. When the mixture cooled to 70–80 °C, 2 mL formaldehyde and 4 mL glacial acetic acid were added and mixed evenly. Ascorbic acid and a multivitamin premix were dissolved in 100 mL distilled water and added when the mixture cooled to ~60 °C. The diet was stirred thoroughly, dispensed into bags, allowed to cool and solidify at room temperature, and stored at 4 °C for further use.

Maize seedlings of the transgenic hybrid Xianda 901ZL and the non-transgenic hybrid Guidan 162 were first raised in the laboratory and transplanted at the two-leaf, one-whorl stage into round plastic pots (20 cm diameter) filled with a 1:1 mixture of local soil and organic substrate to support normal growth. After transplantation, all pots were arranged in a 60 cm × 60 cm equidistant grid layout in the screenhouse to cultivate them for subsequent experiments. The screenhouse was covered with a plastic film at the top to protect the plants from rain, and cultivation was carried out at ambient temperature. In parallel, a cohort of uniform seedlings was transplanted to the Teaching and Research Base of South China Agricultural University (Guangzhou, Guangdong Province, China). The field was planted at 60 cm row spacing and 30 cm within-row spacing to ensure adequate growing space. Both screenhouse and field maize plants were managed using standard agronomic practices, regular irrigation and fertilization, manual weeding, and no insecticide applications throughout the maize growth cycle. To ensure material security, the field site was managed by dedicated personnel with continuous monitoring.

All husbandry complied with institutional biosafety and pest-containment guidelines. No insecticides were applied to experimental plants, and all plant materials and insect cultures were handled under strict containment to prevent escape.

### 2.2. Laboratory Bioassays

In this study, a two-factor experimental design was employed to evaluate the insect resistance of transgenic maize against *S. frugiperda* larvae at different ages. The two main factors were maize genotype (transgenic/non-transgenic) and larval age (1-, 2-, 3-, and 4-day-old larvae, designated L1–L4). Each combination of “genotype × age” constituted one treatment, with five independent replicates per treatment. Each replicate corresponded to a Petri dish or a 24-well plate rearing box.

Young whorl leaves were collected from transgenic and non-transgenic maize plants grown in the screenhouse to the 4–6 leaf stage. In the laboratory, leaf surfaces were gently wiped with moistened sterile gauze to remove dust and debris, then cut into 4 cm^2^ pieces and placed in Petri dishes (60 mm diameter). *S. frugiperda* larvae were gently transferred to the dishes with a fine camel-hair brush. Five *S. frugiperda* larvae were introduced per dish as one replicate, with five replicates established for each larval age group of 1–4-day-old larvae (L1–L4).

Fresh, unpollinated silks were collected from ears at the silking stage. For each Petri dish, 1 g of intact silks was provided as substrate, with an additional 1 g from the same source added once the original silks were completely consumed. Five *S. frugiperda* larvae were introduced per dish as one replicate, with five replicates established for each larval age group of 1–4-day-old larvae.

Kernels at the grain-filling stage were placed individually into the wells of 24-well rearing plates (one kernel per well). For each larval age (L1–L4), two larvae were introduced per well, with five replicate plates per treatment.

All assays were maintained in programmable climate chambers at 26 °C, a 16:8 h light–dark photoperiod, and 70–80% relative humidity. Maize tissue injury and larval survival were monitored daily and photographically documented. Mortality was defined as larvae exhibiting flaccid, blackened bodies with no movement upon gentle probing. The survival (%) was measured as follows:

Survival (%) = (number of live larvae ÷ total larvae) × 100

### 2.3. Screenhouse Potted-Plant Infestation Trials

This study employed a single-factor experimental design to progressively determine the optimal infestation parameters. Initially, the effects of infesting with larvae of different ages were compared to identify the most suitable larval age for infestation. Subsequently, based on this identified age, the infestation density was further optimized. Using a fine camel-hair brush, 20 *S. frugiperda* larvae were gently placed into the whorl funnel of maize plants at the whorl stage (4–6-leaf or 8–10-leaf stage; spaced at 60 cm × 60 cm). Larval ages were 1–4-day-old. Each plant constituted one replicate (*n* = 3 plants per treatment). L2 were inoculated onto maize plants at the 4–6-leaf stage at densities of 10, 20, 30, or 40 larvae per plant. Each plant was treated as one replicate (*n* = 3 plants per treatment). Plant injury was assessed on days 6 and 10 post-infestation by recording the number of notched leaves and the type of whorl damage. The whorl damage types were categorized into four levels with reference to the “Novel 0.0 to 4.0 fall armyworm leaf damage index” proposed by Toepfer et al. [16]: “Windows” (semi-transparent feeding holes appear on the leaves with the leaf margins intact), “Notching” (the whorl leaves exhibit distinct notches from feeding, but the main leaf structure remains intact), “Whorl leaf eaten” (the whorl is heavily fed upon, showing numerous notches or most of the leaf margins consumed), and “Whorl leaf eaten to breakage” (the leaves are destroyed and severed due to feeding). Photographs were taken using a Canon B500 digital camera (Canon Inc., Tokyo, Japan).

### 2.4. Field Infestation Trials

This study employed a single-factor experimental design to progressively determine the optimal infestation parameters. Initially, the infestation efficacy of larvae at different ages was compared to identify the most suitable larval age for infestation. Subsequently, based on this result, the infestation density was further optimized. The experiments were conducted from July to November 2024 at the Teaching and Research Base of South China Agricultural University in Guangzhou, Guangdong Province. The experimental site, along with the broader South China region, is a perennial and recurrent area for fall armyworm occurrence, where stable natural populations are established in the field. All experiments in this study were carried out in compliance with relevant national biosafety management regulations. The experimental maize plants were arranged in the field with a row spacing of 60 cm and a plant spacing of 30 cm. Experimental plants were selected according to the rule of “selecting one plant at every two-plant interval” to determine the locations of test plants. Using a fine camel-hair brush, 20 *S. frugiperda* larvae were gently placed into the whorl funnel of maize plants in the field at the whorl stage (4–6-leaf or 8–10-leaf stage). Larval ages were 1–4-day-old. Each plant constituted one replicate (*n* = 3 plants per treatment). Treatments were randomly assigned to the pre-selected plant locations to ensure consistent environmental conditions across groups. L3 were inoculated onto maize plants at the 4–6-leaf stage at densities of 10, 20, 30, or 40 larvae per plant. Each plant was treated as one replicate (*n* = 3 plants per treatment). All treated plants were laid out in the field following a randomized arrangement, with the rule of selecting one plant at every two-plant interval. Plant injury was assessed on days 7 and 12 post-infestation by recording the number of notched leaves and the type of whorl damage; plants were photographed with a B500 digital camera (Canon Inc., Tokyo, Japan).

### 2.5. Data Analysis

All experimental data were statistically analyzed using Excel 2019. The survival rate of *S. frugiperda* was analyzed for significance using IBM SPSS Statistics 20 software. Data were assessed for normality and homogeneity of variances. Percentage data were subjected to arcsine square root transformation before analysis of variance. For normally distributed data, we used one-way ANOVA to determine the effect of treatment, and means were compared using Tukey’s post hoc test. For the nonnormally distributed data, we used a nonparametric method, the Kruskal–Wallis test, with Bonferroni corrections for multiple comparisons. Different lowercase letters indicate significant differences among treatment groups (*p* < 0.05).

## 3. Results

### 3.1. Laboratory Bioassays of Transgenic Maize Resistance to S. frugiperda

**Figure 1:**
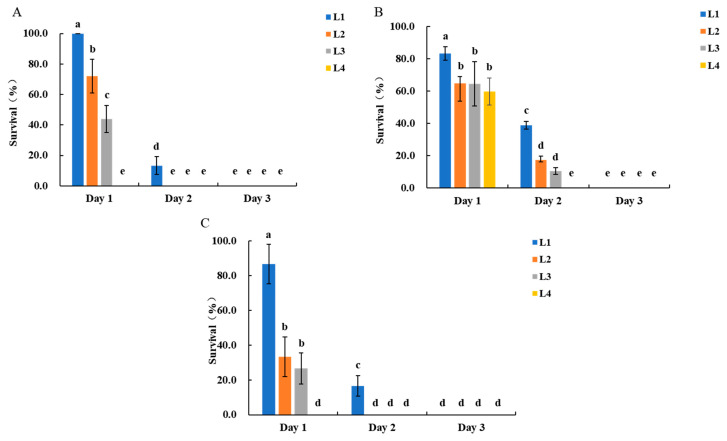
Survival rates of *S. frugiperda* larvae of different ages after feeding on transgenic maize whorl leaves (**A**), silks (**B**), and kernels (**C**). Bars with different lowercase letters above them indicate significant differences between treatments (*p* < 0.05, Tukey’s test). L1–L4 represent 1–4-day-old larvae.

In the leaf assay, larvae feeding on transgenic maize exhibited the following survival rates on day 1 post-infestation: L1 100.0 ± 0.0%, L2 72.0 ± 11.0%, L3 44.0 ± 8.9%, and L4 0.0 ± 0.0%. On day 2, survival rates were 13.3 ± 5.8%, 0.0 ± 0.0%, and 0.0 ± 0.0% for L1, L2, and L3, respectively, while by day 3, no L1 survived (Figure 1A). Correspondingly, transgenic maize leaves showed only slight feeding marks (Figure S1). In contrast, non-transgenic maize plants experienced extensive feeding damage, with large, irregular holes and notched leaves, and in severe cases, only the leaf veins remained, particularly from L2 and L3 (Figure S1).

In the silk assay, survival rates for larvae feeding on transgenic maize were as follows: L1 86.7 ± 11.5%, L2 33.3 ± 11.5%, L3 26.7 ± 11.5%, and L4 0.0 ± 0.0% on day 1 post-infestation. On day 2, survival rates were 16.7 ± 5.8%, 0.0 ± 0.0%, and 0.0 ± 0.0% for L1, L2, and L3, respectively, with no survivors by day 3 (Figure 1B). Correspondingly, transgenic maize silks showed almost no damage, maintaining intact structure (Figure S2). In contrast, non-transgenic maize silks experienced substantial damage, with large sections consumed and only basal remnants remaining.

In the kernel assay, survival rates on day 1 post-infestation for larvae feeding on transgenic maize were as follows: L1 83.3 ± 4.2%, L2 64.6 ± 11.0%, L3 64.6 ± 13.7%, and L4 59.7 ± 8.4%. On day 2, survival rates were 38.9 ± 2.4%, 17.4 ± 1.2%, 10.4 ± 2.1%, and 0.0 ± 0.0% for L1, L2, L3, and L4, respectively, with no survivors of L1, L2, or L3 by day 3 (Figure 1C). Correspondingly, transgenic maize kernels showed minimal damage, maintaining nearly intact structures (Figure S3). In contrast, non-transgenic maize kernels exhibited extensive feeding damage, with large depressions and perforations on the surface (Figure S3).

### 3.2. Potted-Plant Infestation Trials in Screenhouse for Assessing Transgenic Maize Resistance to S. frugiperda

#### 3.2.1. Optimization of Larval Age for Infestation Protocols

**Figure 2:**
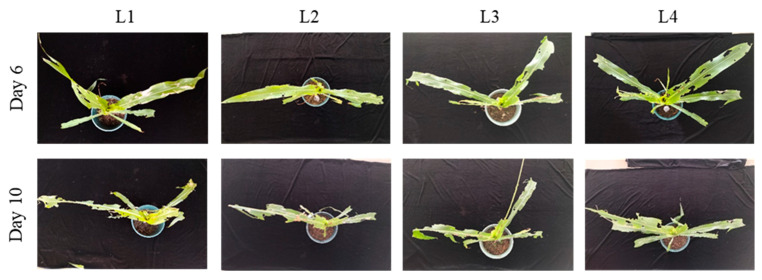
Damage caused by *S. frugiperda* larvae of different ages on non-trangenic maize at the whorl stage (4–6 leaves). This figure shows the damage to maize at the whorl stage caused by larvae of different ages (1–4 days) on days 6 and 10 post-infestation. The amount of leaf notching and the condition of the whorl leaf are shown, with increasing damage observed as larval age increases.

At the 4–6-leaf stage, on day 6 post-infestation, the mean number of notched leaves was 2.7 ± 0.6 for L1 and 4.6 ± 0.6 for L2, with a significant difference between the two [F (3, 8) = 10.00; *p* < 0.05]. No significant differences were observed among the L2, L3, and L4 treatments (Table 1). In terms of whorl-injury types, L1 caused only minor notching and small perforations in the whorl leaves, whereas L2–L4 produced progressively larger notched areas and all resulted in whorl-leaf breakage (Figure 2). On day 10, the number of notched leaves was 3.7 ± 0.6 for L1 and 5.6 ± 0.6 for L2, again showing a significant difference [F (3, 8) = 9.58; *p* < 0.05], while no significant differences were found among the L2–L4 treatments (Table 1). At this stage, all larval ages caused severe injury, including extensive notching. In particular, some leaves suffered a high level of injury, with the mesophyll tissue completely consumed, leaving only the veins intact (Figure 2).

**Figure 3:**
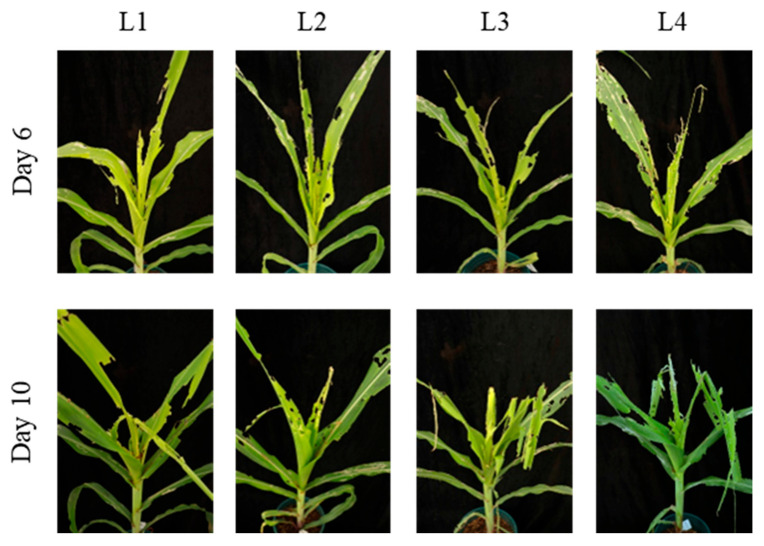
Damage caused by *S. frugiperda* larvae of different ages on non-transgenic maize at the whorl stage (8–10 leaves). This figure shows the damage to maize at the whorl stage (8–10 leaves) caused by larvae of different ages on days 6 and 10 post-infestation. The increasing damage as larval age increases is visually represented.

At the 8–10-leaf stage, on day 6 post-infestation, the mean number of notched leaves was 3.3 ± 0.6 for L1 and 5.3 ± 0.6 for L2, with a significant difference between the two [F (3, 8) = 20.25; *p* < 0.05]; no significant differences were detected among the L2, L3, and L4 treatments (Table 2). In terms of whorl-injury categories, L1 produced minor notching and some perforations in whorl leaves, while L2–L4 caused substantially greater notching, with whorl leaves often reduced to only veins (Figure 3). On day 10, the number of notched leaves was 4.7 ± 0.6 for L1 and 7.0 ± 1.0 for L2, with significant differences between the two [F (3, 8) = 14.44; *p* < 0.05], but no significant differences among L2–L4 treatments (Table 2). At this point, severe damage occurred in all treatments, with large notched areas; some leaves were extensively defoliated, leaving only the midrib intact, and whorl leaves often broken (Figure 3).

#### 3.2.2. Optimization of Larval Density for Infestation Protocols in the Screenhouse

**Figure 4:**
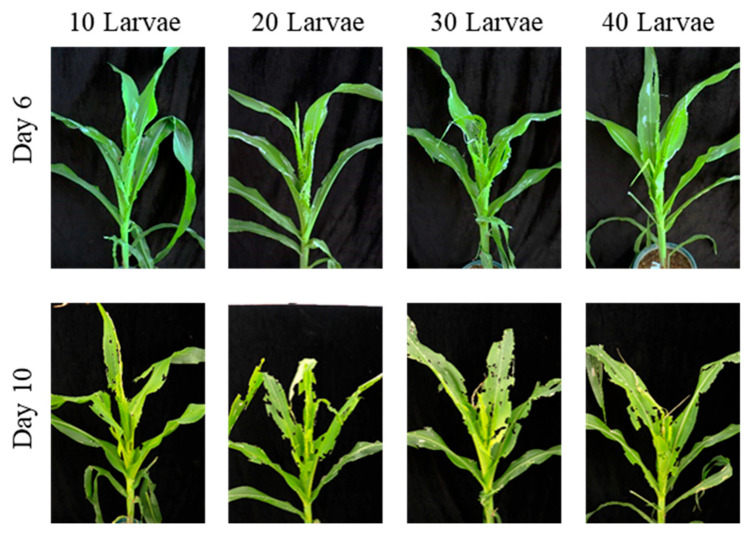
Damage caused by different numbers of 2-day-old *S. frugiperda* larvae on non-transgenic maize at the whorl stage (4–6 leaves). This figure shows how different numbers of 2-day-old larvae (10, 20, 30, 40 larvae per plant) affect maize at the whorl stage. Damage increases with higher larval density, with more extensive leaf notching and whorl-leaf breakage as the larval density increases.

On day 6 post-infestation, the mean number of notched leaves was 2.3 ± 0.6 for the 10 larvae treatment and 3.7 ± 0.6 for the 20 larvae treatment, with a significant difference between the two [F (3, 8) = 9.22; *p* < 0.05]. No significant differences were observed among the 20, 30, and 40 larvae treatments (Table 3). In terms of whorl-injury types, the 10 larvae treatment caused only minor notching, with slight feeding on the whorl leaves and small perforations. The 20 larvae treatment resulted in a significant increase in notched leaves, with more extensive feeding and larger perforations in the whorl leaves. The 30 larvae treatment caused more severe damage, with increased notching and extensive feeding in the whorl. The 40 larvae treatment led to the most severe damage, with many notched leaves and whorl-leaf breakage (Figure 4). On day 10 post-infestation, the number of notched leaves was 3.3 ± 0.6 for the 10 larvae treatment and 5.3 ± 0.6 for the 20 larvae treatment, with a significant difference between the two [F (3, 8) = 8.00; *p* < 0.05]. In terms of whorl injury, all treatments showed severe damage, with extensive notching and large areas of feeding, leaving only the midrib in some leaves. Whorl-leaf breakage was observed in all treatments, and the extent of damage increased with larval density (Figure 4).

### 3.3. Field Infestation Trials for Assessing Transgenic Maize Resistance to S. frugiperda

#### 3.3.1. Comparison of Different Larval Ages for Infestation Protocols

**Figure 5:**
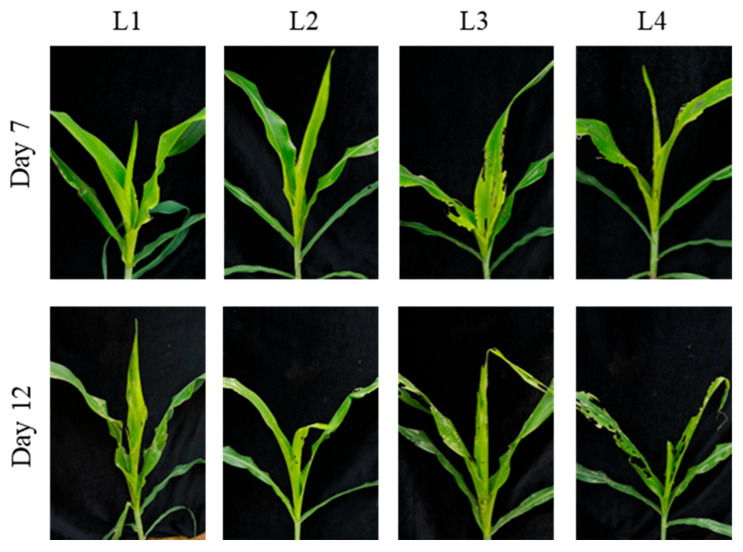
Damage caused by *S. frugiperda* larvae of different ages on non-transgenic maize at the whorl stage (4–6 leaves) in field conditions. This figure illustrates the damage caused by larvae of different ages (1–4 days) on maize in the field. On day 7 and day 12 post-infestation, the damage increases with larval age, showing notching and perforations, with whorl-leaf breakage in more mature larvae.

At the 4–6-leaf stage, on day 7 post-infestation, the mean number of notched leaves was 0.3 ± 0.6 for the L1 treatment, 2.0 ± 0.0 for the L2 treatment, and 3.0 ± 0.0 for L3, with significant differences between L1 and L2, and L1 and L3 treatments [F (3, 8) = 32.67; *p* < 0.05]. No significant differences were observed between the L3 and L4 treatments (Table 4). In terms of whorl-injury types, the L1 and L2 treatments caused light damage, with small notches and slight perforations in the whorl leaves. The L3 and L4 treatments caused progressively more extensive damage, with increased notching and larger perforations in the whorl, resulting in whorl-leaf breakage (Figure 5). By day 12, the number of notched leaves was 2.7 ± 0.6 for L2 and 4.6 ± 0.6 for L3, with a significant difference between the two [F (3, 8) = 11.11; *p* < 0.05], while no significant differences were observed between L1 and L2, or L3 and L4 treatments (Table 4). Whorl damage also increased significantly, with the L1 and L2 treatments showing more notching and slight perforations, while L3 and L4 treatments caused severe notching, large holes, and whorl-leaf breakage (Figure 5).

**Figure 6:**
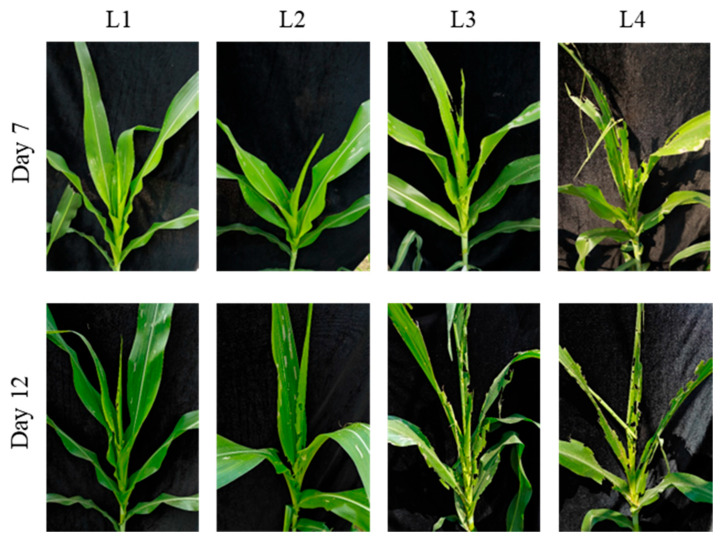
Damage caused by *S. frugiperda* larvae of different ages on maize at the whorl stage (8–10 leaves) in field conditions. This figure shows the damage caused by larvae of different ages (1–4 days) on maize at the 8–10 leaf stage in field conditions. The damage increases over time, with more notching and whorl-leaf breakage as larval age increases.

At the 8–10-leaf stage, on day 7 post-infestation, the mean number of notched leaves for the L1, L2, L3, and L4 treatments was 0.0 ± 0.0, 1.6 ± 0.6, 3.7 ± 0.6, and 4.7 ± 0.6, respectively, with significant differences observed between all treatments [H (3) = 10.34; *p* < 0.05] (Table 5). In terms of whorl-injury types, the L1 treatment showed mostly “windows” (tissue loss without notching), while the L2 treatment exhibited both “windows” and some notching. The L3 treatment caused slight notching, with feeding on the whorl leaves and small holes, while the L4 treatment caused severe notching, large holes, and complete whorl-leaf breakage, leaving only veins (Figure 6). On day 12, the number of notched leaves was 3.3 ± 0.6 for the L1 treatment, 4.0 ± 0.0 for L2, 5.3 ± 0.0 for L3, and 5.6 ± 0.6 for L4, with no significant differences observed between L1 and L2, or L3 and L4 treatments, but with a significant difference between L2 and L3 treatments [H (3) = 9.52; *p* < 0.05] (Table 5). Whorl damage further increased, with the L1 treatment showing mild damage, the L2 treatment showing further notching and larger holes, and the L3 and L4 treatments causing severe damage, including large notches and whorl-leaf breakage (Figure 6).

#### 3.3.2. Optimization of Larval Density for Infestation Protocols in the Field

**Figure 7:**
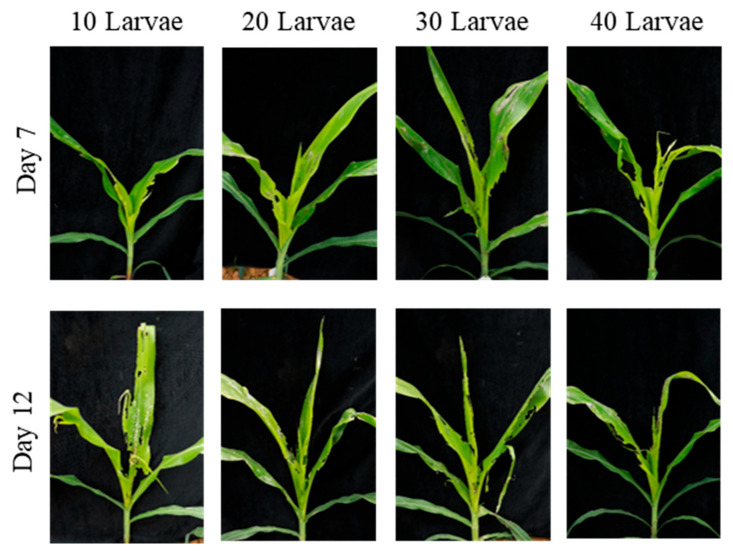
Damage caused by different numbers of 3-day-old *S. frugiperda* larvae on non-transgenic maize at the whorl stage (4–6 leaves) in field conditions. This figure shows the damage caused by different numbers of 3-day-old larvae (10, 20, 30, 40 larvae per plant) on maize at the whorl stage. The damage increases with the number of larvae, showing more notching and extensive feeding as the density increases.

On day 7 post-infestation, the mean number of notched leaves was 2.0 ± 0.0 for the 20 larvae treatment and 3.7 ± 0.6 for the 30 larvae treatment, with a significant difference between the two [H (3) = 9.45; *p* < 0.05]. No significant differences were observed between the 10 larvae and 20 larvae treatments, or between the 30 larvae and 40 larvae treatments (Table 6). In terms of whorl-injury types, the 10 larvae treatment caused minor notching, with slight feeding on the whorl leaves and small perforations. The 20 larvae treatment showed an increase in the area of leaf notching, with the whorl leaves exhibiting more extensive feeding and larger perforations. The 30 larvae treatment caused more severe damage, with increased notching and extensive feeding in the whorl. The 40 larvae treatment led to the most severe damage, with many notched leaves and whorl-leaf breakage (Figure 7). On day 12, the number of notched leaves was 3.3 ± 0.6 for the 20 larvae treatment and 5.0 ± 0.0 for the 30 larvae treatment, with a significant difference between the two [H (3) = 9.97; *p* < 0.05]. No significant differences were found between the 10 larvae and 20 larvae treatments, or between the 30 larvae and 40 larvae treatments (Table 6). In terms of whorl damage, the 10 larvae treatment caused an increase in the number of notched leaves and more extensive feeding, with some leaves showing large notches and whorl-leaf breakage. The 20, 30, and 40 larvae treatments resulted in severe damage, with large notches on the leaves and extensive feeding, leading to whorl-leaf breakage (Figure 7).

## 4. Discussion

The fall armyworm (*S. frugiperda*) is a globally significant agricultural pest native to the tropical and subtropical Americas. Research on transgenic maize resistance to *S. frugiperda* has made notable progress. For instance, Abel et al. [17] evaluated the resistance of eight maize genotypes native to Ecuador through field planting and artificial inoculation, identifying four genotypes with moderate resistance. Moscardini et al. [18] assessed the resistance of six Bt maize lines to *S. frugiperda* through field trials and found that Bt maize expressing the Cry1F, Cry1A.105, Cry2Ab2, and Vip3Aa20 proteins exhibited high resistance to the pest. However, these studies did not standardize the larval age and density for infestation, which may have affected the experimental outcomes and led to longer testing periods. In contrast, the infestation protocol proposed in this study clarifies specific operational parameters. This protocol allows for stable and reliable data within 10–12 days, significantly improving evaluation efficiency and reducing experimental costs, including labor and transportation for field access, diet and manpower for insect rearing, etc. The standardized procedure enhances result reproducibility and facilitates the comparison of data across different laboratories or field trials. It should be noted that the higher infestation densities recommended in this study (e.g., 30 third-instar larvae per plant in field trials) were not designed to simulate natural pest population dynamics, but rather to achieve high efficiency and strong discriminatory power in resistance screening. In resistance evaluation, moderately increasing infestation pressure can accelerate the expression of damage symptoms, shorten the evaluation period, and enhance the differentiation of resistance phenotypes among different genotypes. This approach has been adopted in similar resistance assessment systems [19]. Additionally, under field conditions, the initial infestation level may naturally decline due to factors such as predation and operational losses. Therefore, the designated initial density is intended to ensure that, even after these inevitable reductions, a sufficient number of larvae remain to exert effective and consistent selection pressure on the crops, thereby guaranteeing the stability and reliability of the resistance evaluation. In summary, the present protocol is primarily suitable for rapid, standardized phenotyping of resistance in breeding screening and comparative studies. If the research objective is ecological risk assessment or simulation of natural infestation processes, it is recommended to use infestation densities closer to actual field population levels.

During the study, it was observed that, compared to screenhouse trials, a higher larval density and longer infestation period were required in the field to achieve the same level of maize injury. This difference is primarily due to environmental factors: the screenhouse environment is more controlled, providing optimal conditions for *S. frugiperda* growth. In contrast, field conditions are more influenced by natural climate factors (such as diurnal temperature variations, rainfall, and wind), which may reduce larval activity and delay feeding processes [20,21]. Additionally, field trials are exposed to natural enemies like stink bugs, ladybirds, and various parasitoid wasps, which may limit the movement of *S. frugiperda* larvae and affect the resistance evaluation [22,23,24]. Therefore, when assessing the resistance of transgenic maize to *S. frugiperda*, it is essential to consider the differences between screenhouse and field environments in relation to the study objectives, in order to devise accurate pest control strategies.

While the infestation method established in this study is highly efficient, it still has certain limitations. Firstly, the experiments involved only two maize varieties. Differences in nutritional composition and leaf morphological traits across varieties can significantly influence larval feeding behavior [25,26]. Future studies should therefore incorporate a broader range of genetically diverse maize materials, including transgenic and non-transgenic varieties with different genetic backgrounds, to validate the applicability of this method across diverse varietal systems and to elucidate how plant trait–insect behavior interactions affect resistance assessment outcomes. Secondly, the field trials were conducted solely in South China, where climatic and ecological conditions differ from those in other major maize-producing regions of China, such as the Southwest, the middle and lower reaches of the Yangtze River, and areas at varying altitudes. To verify and enhance the regional applicability of the method, follow-up work should involve multi-location, multi-year replicated trials in these key ecological zones, systematically monitoring the influence of environmental factors (e.g., temperature, humidity, rainfall) on infestation efficacy and damage severity, thereby establishing a more broadly applicable standardized protocol. Despite these limitations, this study provides a relatively accurate and specific method for evaluating the damage caused by *S. frugiperda* to maize, offering valuable insights for resistance assessments of transgenic maize both indoors and in the field. It also provides important guidance for the development of standardized infestation protocols for *S. frugiperda*.

## Figures and Tables

**Table 1:** Damage caused by *S. frugiperda* larvae of different ages on non-transgenic maize at the whorl stage (4–6 leaves) in screenhouse conditions.

Larval Age	Day 6		Day 10	
	Number of Notched Leaves	Whorl Injury Type	Number of Notched Leaves	Whorl Injury Type
L1	2.7 ± 0.6 b	Whorl leaf eaten	3.7 ± 0.6 b	Whorl leaf eaten to breakage
L2	4.6 ± 0.6 a	Whorl leaf eaten to breakage	5.6 ± 0.6 a	Whorl leaf eaten to breakage
L3	5.0 ± 1.0 a	Whorl leaf eaten to breakage	6.3 ± 0.6 a	Whorl leaf eaten to breakage
L4	5.7 ± 0.6 a	Whorl leaf eaten to breakage	6.6 ± 0.6 a	Whorl leaf eaten to breakage

The data in the table are presented as mean ± standard error, and those in the same column followed by different lowercase letters are significantly different (*p* < 0.05, Tukey’s test).

**Table 2:** Damage caused by *S. frugiperda* larvae of different ages on non-transgenic maize at the whorl stage (8–10 leaves) in screenhouse conditions.

Larval Age	Day 6		Day 10	
	Number of Notched Leaves	Whorl Injury Type	Number of Notched Leaves	Whorl Injury Type
L1	3.3 ± 0.6 b	Whorl leaf eaten to breakage	4.7 ± 0.6 b	Whorl leaf eaten to breakage
L2	5.3 ± 0.6 a	Whorl leaf eaten to breakage	7.0 ± 1.0 a	Whorl leaf eaten to breakage
L3	6.3 ± 0.6 a	Whorl leaf eaten to breakage	7.3 ± 0.6 a	Whorl leaf eaten to breakage
L4	6.7 ± 0.6 a	Whorl leaf eaten to breakage	8.3 ± 0.6 a	Whorl leaf eaten to breakage

The data in the table are presented as mean ± standard error, and those in the same column followed by different lowercase letters are significantly different (*p* < 0.05, Tukey’s test).

**Table 3:** Damage caused by different numbers of 2-day-old *S. frugiperda* larvae on non-transgenic maize at the whorl stage (4–6 leaves).

Number of Larvae	Day 6		Day 10	
	Number of Notched Leaves	Whorl Injury Type	Number of Notched Leaves	Whorl Injury Type
10	2.3 ± 0.6 b	Whorl leaf eaten	3.3 ± 0.6 b	Whorl leaf eaten to breakage
20	3.7 ± 0.6 a	Whorl leaf eaten	5.3 ± 0.6 a	Whorl leaf eaten to breakage
30	4.0 ± 0.0 a	Whorl leaf eaten	5.3 ± 0.6 a	Whorl leaf eaten to breakage
40	4.3 ± 0.6 a	Whorl leaf eaten to breakage	6.0 ± 1.0 a	Whorl leaf eaten to breakage

The data in the table are presented as mean ± standard error, and those in the same column followed by different lowercase letters are significantly different (*p* < 0.05, Tukey’s test).

**Table 4:** Damage caused by *S. frugiperda* larvae of different ages on non-transgenic maize at the whorl stage (4–6 leaves) in field conditions.

Larval Age	Day 7		Day 12	
	Number of Notched Leaves	Whorl Injury Type	Number of Notched Leaves	Whorl Injury Type
L1	0.3 ± 0.6 c	Windows/Notching	2.3 ± 0.6 b	Whorl leaf eaten
L2	2.0 ± 0.0 b	Windows/Notching	2.7 ± 0.6 b	Whorl leaf eaten to breakage
L3	3.0 ± 0.0 a	Whorl leaf eaten	4.6 ± 0.6 a	Whorl leaf eaten to breakage
L4	3.3 ± 0.6 a	Whorl leaf eaten	5.0 ± 1.0 a	Whorl leaf eaten to breakage

The data in the table are presented as mean ± standard error, and those in the same column followed by different lowercase letters are significantly different (*p* < 0.05, Tukey’s test).

**Table 5:** Damage caused by *S. frugiperda* larvae of different ages on non-transgenic maize at the whorl stage (8–10 leaves) in field conditions.

Larval Age	Day 7		Day 12	
	Number of Notched Leaves	Whorl Injury Type	Number of Notched Leaves	Whorl Injury Type
L1	0.0 ± 0.0 d	Windows	3.3 ± 0.6 b	Whorl leaf eaten
L2	1.6 ± 0.6 c	Windows/Notching	4.0 ± 0.0 b	Whorl leaf eaten to breakage
L3	3.7 ± 0.6 b	Whorl leaf eaten	5.3 ± 0.0 a	Whorl leaf eaten to breakage
L4	4.7 ± 0.6 a	Whorl leaf eaten to breakage	5.6 ± 0.6 a	Whorl leaf eaten to breakage

The data in the table are presented as mean ± standard error, and those in the same column followed by different lowercase letters are significantly different (*p* < 0.05, Kruskal–Wallis test followed by Bonferroni correction).

**Table 6:** Damage caused by different numbers of 3-day-old *S. frugiperda* larvae on non-transgenic maize at the whorl stage (4–6 leaves).

Number of Larvae	Day 7		Day 12	
	Number of Notched Leaves	Whorl Injury Type	Number of Notched Leaves	Whorl Injury Type
10	1.7 ± 0.6 b	Whorl leaf eaten	3.0 ± 0.0 b	Whorl leaf eaten to breakage
20	2.0 ± 0.0 b	Whorl leaf eaten	3.3 ± 0.6 b	Whorl leaf eaten to breakage
30	3.7 ± 0.6 a	Whorl leaf eaten/eaten to breakage	5.0 ± 0.0 a	Whorl leaf eaten to breakage
40	3.7 ± 0.6 a	Whorl leaf eaten/eaten to breakage	5.7 ± 0.6 a	Whorl leaf eaten to breakage

The data in the table are presented as mean ± standard error, and those in the same column followed by different lowercase letters are significantly different (*p* < 0.05, Kruskal–Wallis test followed by Bonferroni correction).

## Data Availability

The original contributions presented in this study are included in the article/Supplementary Material. Further inquiries can be directed to the corresponding author.

## Supplementary Materials

The following supporting information can be downloaded at: https://www.mdpi.com/article/10.3390/insects17020136/s1, Figure S1. Damage caused by *S. frugiperda* larvae of different ages on maize whorl leaves. Maize whorls at the 4–6 leaf stage were infested with larvae at 1–4 days post-hatching, and damage severity was recorded daily. It depicts the visual differences in leaf damage between transgenic and non-transgenic maize over time. The abbreviation GM stands for genetically modified maize, while non-GM refers to non-genetically modified maize. L1–L4 represent 1–4-day-old larvae; Figure S2. Damage caused by *S. frugiperda* larvae of different ages on maize silks. Silks from the silking stage were infested with larvae at 1–4 days post-hatching and observed daily. All larval ages caused severe feeding damage on non-Bt silks, with extensive consumption and only basal parts remaining in some cases. Conversely, Bt silks remained nearly intact with almost no signs of feeding. L1–L4 represent 1–4-day-old larvae; Figure S3. Damage caused by *S. frugiperda* larvae of different ages on maize kernels. Maize kernels were infested with two larvae (1–4 days post-hatching) per kernel and inspected daily. The extent of kernel damage is visualized, comparing the effects on transgenic and non-transgenic maize. L1–L4 represent 1–4-day-old larvae.
