# Supplementary material for: Rapid Artificial Infestation Method for Assessing Fall Armyworm (Spodoptera frugiperda) Damage on Maize
Source: Insects. 2026 Jan 24;17(2):136. doi: 10.3390/insects17020136 (PMC12940814; doi:10.3390/insects17020136)
Supplement: Supplementary file 1 [file insects-17-00136-s001.zip › insects-4044310-supplementary.pdf]

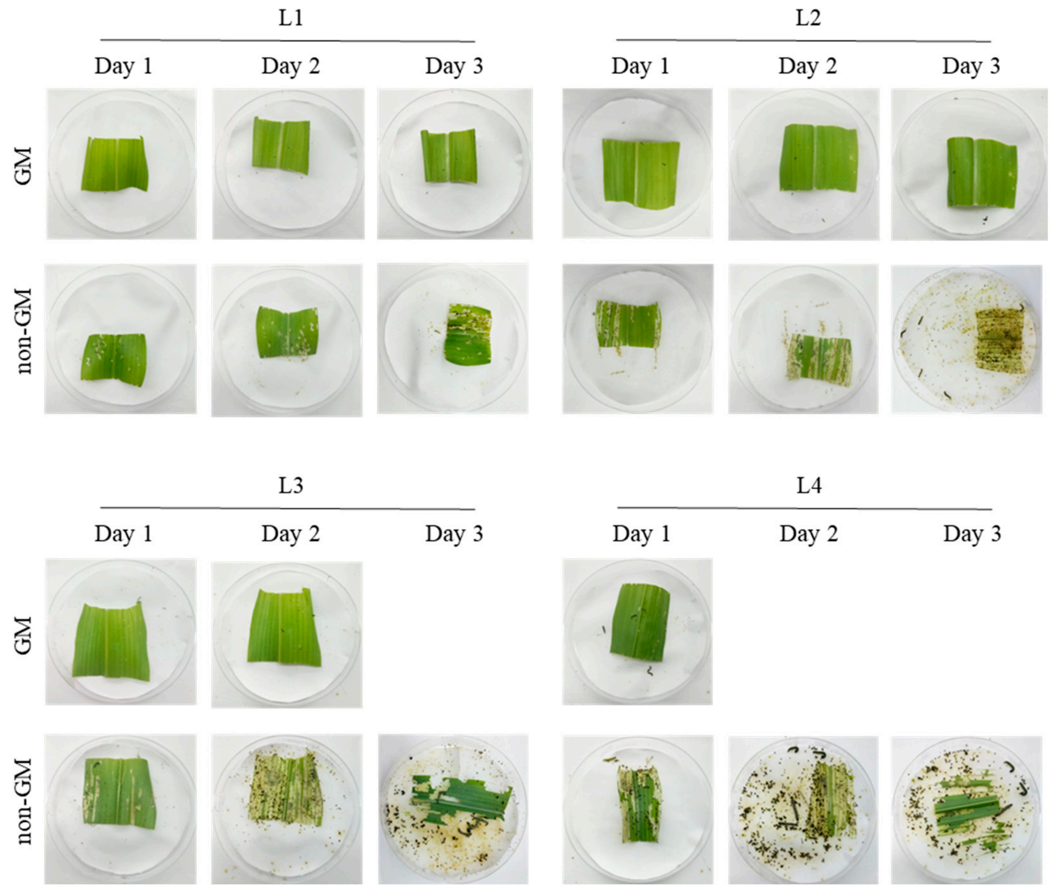

Figure S1. Damage caused by *S. frugiperda* larvae of different ages on maize whorl leaves. Maize whorls at the 4–6 leaf stage were infested with larvae at 1–4 days post-hatching, and damage severity was recorded daily. It depicts the visual differences in leaf damage between transgenic and non-transgenic maize over time. The abbreviation GM stands for genetically modified maize, while non-GM refers to non-genetically modified maize. L1–L4 represent 1–4-day-old larvae.

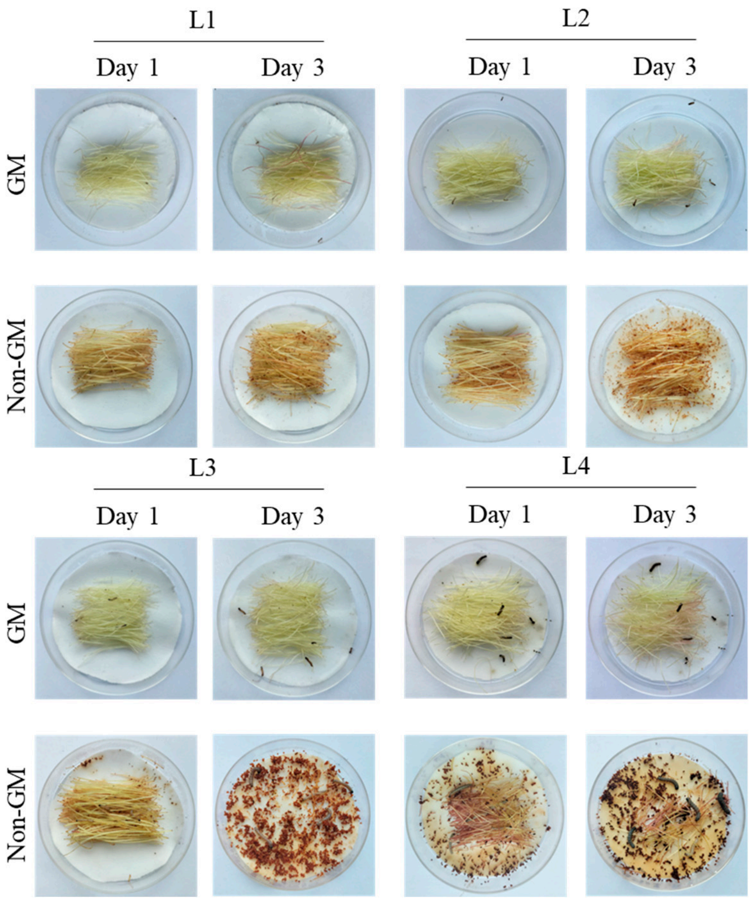

Figure S2. Damage caused by *S. frugiperda* larvae of different ages on maize silks. Silks from the silking stage were infested with larvae at 1–4 days post-hatching and observed daily. All larval ages caused severe feeding damage on non-Bt silks, with extensive consumption and only basal parts remaining in some cases. Conversely, Bt silks remained nearly intact with almost no signs of feeding. L1–L4 represent 1–4-day-old larvae.

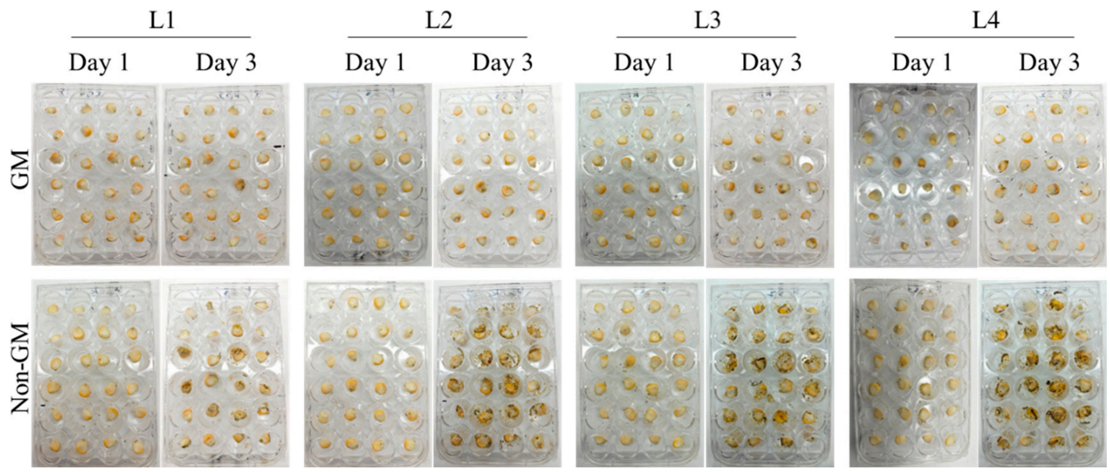

Figure S3. Damage caused by *S. frugiperda* larvae of different ages on maize kernels. Maize kernels were infested with two larvae (1–4 days post-hatching) per kernel and inspected daily. The extent of kernel damage is visualized, comparing the effects on transgenic and non-transgenic maize. L1–L4 represent 1–4-day-old larvae.
